# CDK12 inactivation across solid tumors: an actionable genetic subtype

**DOI:** 10.18632/oncoscience.481

**Published:** 2019-05-10

**Authors:** Catherine H. Marshall, Eddie L. Imada, Zhuojun Tang, Luigi Marchionni, Emmanuel S. Antonarakis

**Affiliations:** ^1^ Sidney Kimmel Comprehensive Cancer Center, Johns Hopkins University School of Medicine, Baltimore, Maryland, USA; ^2^ Departamento de Bioquimica e Imunologia, Universidade Federal de Minas Gerais, Belo Horizonte, Minas Gerais, Brazil; ^3^ Sidney Kimmel Comprehensive Cancer Center and Center for Computational Genomics, Johns Hopkins School of Medicine, Baltimore, Maryland, USA

**Keywords:** prostate cancer, CDK12, genetics, immunotherapy, biomarkers

## Abstract

Inactivating *CDK12* alterations have been reported in ovarian and prostate cancers and may have therapeutic implications; however, the prevalence of these mutations across other cancer types is unknown. We searched the cBioPortal and GENIE Project (public release v4.1) databases for cancer types with > 200 sequenced cases, that included patients with metastatic disease, and in which the occurrence of at least monoallelic *CDK12* alterations was > 1%. The prevalence of at least monoallelic *CDK12* mutations was highest in bladder cancer (3.7%); followed by prostate (3.4%), esophago-gastric (2.1%) and uterine cancers (2.1%). Biallelic *CDK12* inactivation was highest in prostate cancer (1.8%), followed by ovarian (1.0%) and bladder cancers (0.5%). These results are the first (to our knowledge) to estimate the prevalence of monoallelic and biallelic *CDK12* mutations across multiple cancer types encompassing over 15,000 cases.

## INTRODUCTION

Inactivating *CDK12* alterations have been reported in ovarian and prostate cancers; however, the prevalence of these mutations across all cancer types is unknown [1]. While *CDK12* was initially thought to be involved in homologous-recombination DNA repair, emerging data suggest a unique role of this gene in DNA replication-associated repair. To this end, it has been suggested that inactivating *CDK12* mutations lead to widespread focal genomic duplications that generate gene fusion-induced neoantigens and favorable responses to immune-checkpoint blockade therapy using PD-1 inhibitors [2]. Given this potentially actionable molecular subtype, we sought to determine the prevalence of monoallelic and biallelic *CDK12* alterations across tumor types.

## RESULTS

**Figure 1:**
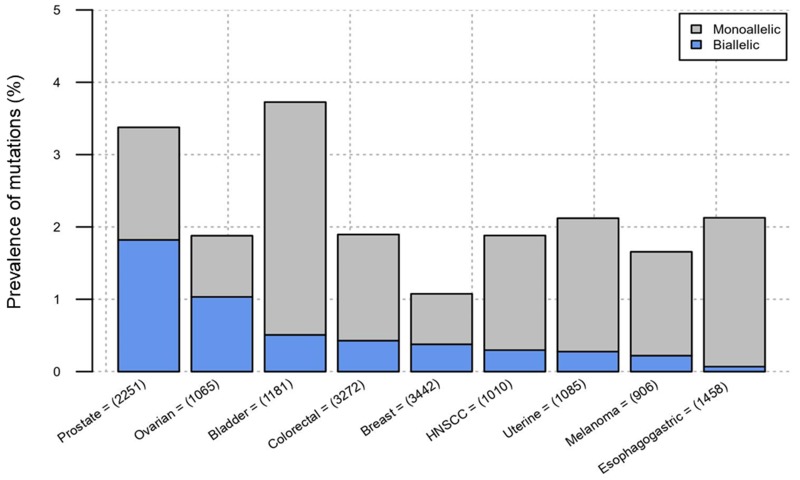
Prevalence of *CDK12* mutations across 9 cancer types

Datasets (in cBioPortal and GENIE) from prostate, breast, colorectal, bladder, ovarian, uterine, head-and-neck squamous cell carcinoma, melanoma, and esophago-gastric cancers were included (Table 1); other tumor types did not reach a 1% frequency of *CDK12* alterations. The prevalence of at least monoallelic *CDK12* mutations was highest in bladder cancer (3.7%); followed by prostate (3.4%), esophago-gastric (2.1%) and uterine cancers (2.1%). Biallelic *CDK12* inactivation was highest in prostate cancer (1.8%), followed by ovarian (1.0%) and bladder cancers (0.5%) (Figure 1).

**Table 1:** Datasets publically available from cBioPortal and GENIE Project that were used, by disease group, with overall sample size

Disease	Dataset	Sample Size	Total
**Bladder**	BLCA_TCGA_PAN_CAN_ATLAS_2018	408	1,181
	DFCI-ONCOPANEL-3	69	
	MSK-IMPACT341	95	
	MSK-IMPACT410	326	
	MSK-IMPACT468	143	
	UTUC_MSKCC_2013	84	
	VICC-01-T5A	3	
	VICC-01-T7	53	
**Breast**	BRCA_IGR_2015	216	3,442
	BRCA_MBCPROJECT_WAGLE_2017	157	
	DFCI-ONCOPANEL-3	304	
	MSK-IMPACT341	410	
	MSK-IMPACT410	1,021	
	MSK-IMPACT468	1,076	
	VICC-01-T5A	87	
	VICC-01-T7	171	
**Colorectal**	CRC_MSK_2018	1,134	3,272
	DFCI-ONCOPANEL-3	351	
	MSK-IMPACT341	209	
	MSK-IMPACT410	906	
	MSK-IMPACT468	465	
	VICC-01-T5A	47	
	VICC-01-T7	160	
**Esophagogastric**	DFCI-ONCOPANEL-3	146	1,458
	EGC_MSK_2017	341	
	ESCA_TCGA_PAN_CAN_ATLAS_2018	182	
	MSK-IMPACT341	122	
	MSK-IMPACT410	216	
	MSK-IMPACT468	106	
	STES_TCGA_PUB	288	
	VICC-01-T5A	11	
	VICC-01-T7	46	
**HNSCC**	DFCI-ONCOPANEL-3	83	1, 010
	HNC_MSKCC_2016	151	
	HNSC_TCGA_PAN_CAN_ATLAS_2018	517	
	MSK-IMPACT341	37	
	MSK-IMPACT410	132	
	MSK-IMPACT468	75	
	VICC-01-T5A	6	
	VICC-01-T7	9	
**Melanoma**			906
	MSK-IMPACT341	64	
	MSK-IMPACT410	364	
	MSK-IMPACT468	214	
	VICC-01-T7	140	
	VICC-01-T5A	37	
**Ovarian**	DFCI-ONCOPANEL-3	125	1,065
	MSK-IMPACT341	88	
	MSK-IMPACT410	139	
	MSK-IMPACT468	155	
	OV_TCGA_PUB	489	
	VICC-01-T5A	31	
	VICC-01-T7	38	
**Prostate**	DFCI-ONCOPANEL-3	97	2,251
	MSK-IMPACT341	153	
	MSK-IMPACT410	569	
	MSK-IMPACT468	377	
	PRAD_FHCRC	149	
	PRAD_MICH	61	
	PRAD_MSKCC	194	
	PRAD_SU2C_2015	150	
	PRAD_MSKCC_2017	501	
**Uterine**	DFCI-ONCOPANEL-3	120	1,085
	MSK-IMPACT341	119	
	MSK-IMPACT410	258	
	MSK-IMPACT468	326	
	UCEC_TCGA_PUB	232	
	VICC-01-T7	19	
	VICC-01-T5A	11	

## DISCUSSION

In the era of precision oncology, inactivation of *CDK12* may represent a new molecular subtype with therapeutic implications [6], although the pan-cancer prevalence of this genomic alteration was previously unknown. These results are the first (to our knowledge) to estimate the prevalence of monoallelic and biallelic *CDK12* mutations across nine cancer types encompassing >15,000 cases. This is important as *CDK12* alterations may be implicated in favorable responses to immune checkpoint inhibition, with biallelic alterations theoretically expected to respond better than monoallelic alterations. Prospective clinical trials (e.g. NCT03570619) are now needed to adequately assess this therapeutic hypothesis, and our data could be useful in the design of such trials.

Our results are limited to data that were publicly available. In addition, genotyping and mutation calling are sensitive to several factors, *e.g.* quality of the sample, sequencing depth and platform, and the pipeline used. Additionally, datasets from the GENIE Project revealed overall lower *CDK12* mutation rates than datasets retrieved from cBioPortal. The reason for this is unclear but may include different pipelines with different sensitivity and specificity, artifacts due to DNA damage in sample preparation found in the capture-panels used in the GENIE Project, and differing sample quality (all samples from the GENIE Project were formalin-fixed paraffin-embedded while most from cBioPortal were fresh-frozen samples) [3–5]. Because of this, we hypothesize that our reported prevalences are likely underestimates of the true frequency of these mutations. Nevertheless, our analysis suggests that there are at least nine cancer types with a *CDK12* mutation prevalence between 1-4%, hopefully prompting further exploration of immunotherapy approaches using a basket-trial design. Given the recent FDA-approval of larotrectinib for *NTRK*-altered cancers regardless of histologic type, we envision a similar mode of clinical exploration for *CDK12*-altered tumors.

## METHODS

We searched the cBioPortal [3,4] and GENIE Project (public release v4.1) [5] databases for cancer types with ≥200 sequenced cases, that included patients with metastatic disease, and in which the prevalence of at least monoallelic *CDK12* alterations was ≥1%. Analyses were restricted to datasets containing both *CDK12* mutation and copy-number alteration (CNA) data using hybridization-capture panels from Dana-Farber Cancer Institute, Memorial Sloan-Kettering Cancer Center and Vanderbilt-Ingram Cancer Center. *CDK12* mutations were considered inactivating (i.e. resulting in loss-of-function) in the case of homozygous loss, genomic rearrangements, frameshift or nonsense protein-truncating mutations, splice-site mutations, or missense mutations within the kinase domain. Monoallelic alterations were defined as at least one protein-truncating *CDK12* variant; biallelic alterations were defined as a protein-truncating variant plus a second protein-truncating variant, a kinase domain missense variant, or loss-of-heterozygosity of the wild-type *CDK12* allele. All analyses were performed in R.
